# PROTOCOL: Situational Crime Prevention Measures to Prevent Terrorist Attacks Against Soft Targets and Crowded Places: An Evidence and Gap Map

**DOI:** 10.1002/cl2.70040

**Published:** 2025-04-28

**Authors:** Zoe Marchment, Caitlin Clemmow, Paul Gill

**Affiliations:** ^1^ University College London London UK

## Abstract

This is the protocol for a Campbell systematic review. The objectives are as follows. The EGM has three main objectives: (1) Identify the strength (in terms of evidence quality) and depth (in terms of volume of evidence) of evidence base on the efficacy of situational crime prevention measures in preventing terrorist attacks against soft targets and crowded places. (2) Identify the heterogeneity in the effects of situational crime prevention measures against terrorist attacks and link this to issues related to context and implementation. (3) Identify the mechanisms through which situational crime prevention measures have an effect on terrorist attacks. To achieve these objectives, an EGM will seek out reliable quantitative evidence on effect and qualitative evidence on mechanisms, moderators, implementation and economics. Resultingly, it will be possible to identify research gaps and evidence imbalances to facilitate research investment, identify gaps and topics for new research, and provide a foundation for systematic reviews by showing where sufficient evidence exists for aggregation. The underpinning programme of work will result in the presentation of rigorous empirical research on this topic to help researchers and decision‐makers understand the available evidence.

## 1 Background

### 1.1 Introduction

#### 1.1.1 The Problem, Condition or Issue

Preventing terrorist attacks remains a global priority. In 2023, 66 terrorist groups were responsible for 3350 terrorist attacks. Deaths through terrorism were at their highest level globally since 2017, totalling 8352 (Institute for Economics & Peace. Global Terrorism Index 2024). An increase in target hardening designed to prevent attacks against prominent targets such as officials and the military (Brandt and Sandler 2010) has resulted in an increasing preference to commit attacks against soft targets and crowded places. These types of locations include buildings and open spaces that have limited protective measures in place, such as schools, transport hubs, tourist attractions, sports and leisure venues, bars and restaurants, and places of worship. These types of places are vulnerable to terrorist attacks due to their easy accessibility and lack of security measures.

Consistent with developments in the area of crime control (Cornish and Clarke 1986; Brantingham and Brantingham 1981), one response amongst the academic community in the field of terrorism studies has been to consider the situational qualities of terrorist behaviour—in other words, what terrorists do and how they do it (Horgan 2004). The view of the rational offender is now a mainstay of the criminology literature (Cornish and Clarke 2016), and extensive evidence exists to demonstrate the applicability of rational choice explanations to criminal decision‐making. Offender decision‐making is influenced by opportunities in the immediate environment and reflects everyday behaviour. Simply put, crime is judged to occur when the anticipated benefits outweigh the perceived costs and effort involved (Becker 1968). The emergent evidence base on terrorist decision‐making, in turn, highlights considerable overlaps with what is known about criminal decision‐making on issues like target choice (Gruenewald et al. 2015), weapon choice (Legault and Hendrickson 2009), spatio‐temporal clustering (Braithwaite and Johnson 2015; Tench et al. 2016), the distances travelled to commit a terrorist attack (Marchment and Gill 2019; Marchment et al. 2020), and displacement in terms of target type and weapon use (Hsu and Apel 2015). Like traditional criminals, terrorists are rational in their spatial decision‐making regarding where to target. Their target selection is guided by an inherent logic, influenced by the characteristics of target areas (Marchment and Gill 2019). Terrorist decisions about where to carry out a particular attack can derive from cost–benefit analyses, drawing on prior experience, acquired knowledge (from a variety of sources including but not limited to online research, site visits, and insider knowledge) and information in the immediate attack situation (Gill et al. 2020). Issues such as security measures, avoiding detection, ease of access and escape are often considered at length (Gill 2015).

Because criminal and terrorist decision‐making appears similar, it is worthwhile enquiring whether analogous strategies aimed at preventing and disrupting the onset of these offences also work similarly. The influence of place on the risk of criminal activity is well established within the study of more traditional crime. The field of crime prevention is testament to the vast potential for situationally focused crime prevention approaches, defined as ‘opportunity‐reducing measures that (1) are directed at highly specific forms of crime, (2) involve the management, design and manipulation of the immediate environment in as systematic and permanent a way as possible, and (3) make crime more difficult and risky, or less rewarding and excusable as judged by a wide range of offenders’ (Clarke 2006, 4).

#### 1.1.2 The Intervention

Features of design play a key role in vulnerability to crime at the neighbourhood, street and individual property level (Armitage 2017), and situational factors that increase the risks associated with a criminal opportunity can strongly influence criminal decision‐making. In the same way, terrorists often choose a target that has the perceived fewest risks when compared to all other possibilities. The opportunity to commit an attack depends on finding a suitable target that is insufficiently guarded (Cohen and Felson 1979). The main focus of situational crime prevention (SCP) is to increase the difficulty of committing a crime by altering the physical environment. SCP consists of five prevention mechanisms: increase the effort, increase the risk, reduce rewards, reduce provocations, and remove excuses (Cohen and Felson 1979). Effort is increased via measures such as target hardening, controlling access to facilities, deflecting offenders, and controlling access to the necessary weapons. Risks are increased through measures such as extending guardianship, assisting with natural surveillance, and increasing surveillance. The rewards of an attack can be reduced by concealing or removing potential targets.

Clarke (2006) was the first to argue that SCP could be used to prevent terrorist attacks. Since then, research regarding its application to terrorism has increased. Each type of modus operandi used in a terror attack is dependent on multiple conditions coming together, which offer their own set of environmental conditions. These can be manipulated to influence the terrorist cost–benefit calculus.

For example, in 2019, Mark Domingo was arrested and convicted for plotting to bomb a neo‐Nazi rally in Long Beach, California. In the build‐up to the attack, he had considered multiple other targets. One included a Jewish place of worship. In a conversation with an undercover FBI agent, Domingo noted he was deterred because the area was upscale and had a lot of businesses and security cameras (USA vs. Domingo, 2019). Eric Rudolph conducted the 1996 Centennial Olympic Park bombing in Atlanta. In the days before the bombing, he conducted a reconnaissance of the area. His autobiography notes: ‘Hundreds of security guards and cops patrolled the park. They eyeballed me going through the entrances. But there were no metal detectors, and bags were searched selectively. After sundown, the crowds grew enormous … Security at the park became overwhelmed. They stopped searching for bags altogether, and the entrances flew wide open. I knew then that I could smuggle in a bomb’ (Gill et al. 2020). A jihadist plot involving weapons, hostage‐taking, and explosives decided against targeting a synagogue because they tended to be ‘monitored by loads of security cameras’ (Torres‐Soriano 2021).

However, despite the intuitive appeals of the implementation of SCP measures to prevent terrorism, no synthesis of the evidence base has been undertaken as guidance. This evidence and gap map will provide a comprehensive understanding of what is known (or not) in this topic area about quantitative impact evaluations of such interventions on the rate/severity of all forms of terrorist attacks, value‐for‐money evaluations, and qualitative evidence on how and why such interventions work or not. Interventions can also result in unintended consequences—both negative and positive. Crime displacement is associated with criminal behaviour that is observed elsewhere or at different times because of that intervention, while diffusion of benefits sees a reduction in crime among nearby locations and times that were not targeted by the intervention. As such, we will also examine five types of displacement that could occur subsequent to an intervention, (a) spatial displacement: a change in the location of attack, (b) temporal displacement: change of activity by the time of occurrence, (c) target displacement: attack against a different target, (d) tactical displacement: the perpetrator adopts a separate attack type, and (e) perpetrator displacement: when opportunities for a new type of terrorist offender occur (Barr and Pease 1990). Additionally, we are interested in identifying and reporting issues related to populations experiencing inequities as a result of the SCP intervention (e.g., certain populations may be adversely impacted via biased formal surveillance programmes, or certain SCP measures may evoke more terrorist attacks in certain settings).

#### 1.1.3 Why It Is Important to Develop the EGM

Across different national contexts, government strategy documents to protect soft targets and crowded places refer to the need for protective security and SCP measures without making reference to accompanying evidence to suggest which measures might work best in a given place. For example, Australia's Crowded Place strategy suggests ‘installing barriers and gates’, while New Zealand's strategy refers to target hardening measures like ‘fences and electronic access control’ without reference to evidence and without consideration that crowded places may differ from one another on many dimensions and that each measure might not be equally applicable to each location. This study is also necessary because of the lack of available guidance on how to conduct an evaluation of these measures. Again, referencing Australia's Crowded Place strategy, owners and operators of crowded places are encouraged to evaluate their situational measures without any accompanying guidance on what appropriate and robust evaluations would look like. The value of an evidence and gap map lies in its ability to provide a clear, organized overview of the existing research landscape, highlight where further work is needed, and facilitate evidence‐based decision‐making and policy formulation. It is an efficient tool for ensuring that research efforts are targeted and that resources are used where they are most needed. The primary function of our EGM is to inform future funding priorities, such as further primary studies or systematic reviews. As well as making the existing evidence base discoverable and useable for decision‐makers aiming to protect soft targets, it will also set the thresholds for evidentiary quality for decision‐makers to assess newly emergent research studies. A secondary function of our EGM is to identify where evidence is lacking.

To date, although there are literature reviews with much wider foci than outcome evaluations, no EGM nor systematic review exists specifically in this area of terrorism prevention (e.g., Freilich et al. 2018), although one does exist in the area of criminal justice interventions for preventing radicalization (Sydes et al. 2023). Systematic reviews exist on situational interventions such as various measures (Grove et al. 2012), CCTV (Piza et al. 2019), and crime prevention through environmental design (Casteel and Peek‐Asa 2000). However, the outcomes are all related to other forms of crime, such as burglary and robbery, and not terrorism. SCP is designed to be directed at specific types of behaviour, and it is in this form of application that it works best. Although other reviews exist, they are not designed to ascertain the effectiveness of such measures in the specific context of all forms of terrorist attacks. Importantly, the EGM will also provide the ability to filter across a range of ideological influences to establish whether certain interventions work dissimilarly across different ideologies themselves and may perceive costs and benefits in different ways.

## 2 Objectives

The EGM has three main objectives:

1. Identify the strength (in terms of evidence quality) and depth (in terms of volume of evidence) of the evidence base on the efficacy of SCP measures in preventing terrorist attacks against soft targets and crowded places.
2. Identify the heterogeneity in the effects of SCP measures against terrorist attacks and link this to issues related to context and implementation.
3. Identify the mechanisms through which SCP measures have an effect on terrorist attacks.

To achieve these objectives, an EGM will seek out reliable quantitative evidence on effect and qualitative evidence on mechanisms, moderators, implementation and economics. Resultingly, it will be possible to identify research gaps and evidence imbalances to facilitate research investment, identify gaps and topics for new research, and provide a foundation for systematic reviews by showing where sufficient evidence exists for aggregation. The underpinning programme of work will result in the presentation of rigorous empirical research on this topic to help researchers and decision‐makers understand the available evidence.

## 3 Methods

### 3.1 Evidence and Gap Map: Definition and Purpose

EGMs are a visual tool that systematically organizes and presents existing research evidence on a specific topic or area of interest, in this case, the efficacy of SCP measures to prevent terrorist attacks on soft targets or crowded places. EGMs typically map the relationships between interventions (or practices) and outcomes, highlighting where robust evidence exists and where there are gaps in the research. Our EGM will additionally provide information on the contexts, settings, and implementation issues associated with a range of SCP measures. EGMs are built from systematic searches of the literature, following a structured process to identify and categorize all relevant studies. They often take the form of matrices, grids, or interactive charts where interventions are displayed along one axis and outcomes along another, making it easy to spot well‐researched areas and evidence gaps. EPPI‐Mapper will be used to create the EGM and visualization. EPPI‐Mapper is a standalone tool originally designed for the Campbell Collaborations Evidence and Gap Maps, now freely available to EPPI‐Reviewer users. EPPI‐Mapper takes input from coding in EPPI‐Reviewer to create a ‘map’ of research evidence. The output is an interactive HTML file hosted freely by the EPPI platform. In our case, SCP interventions will be displayed along one axis and outcomes along another, making it easy to spot well‐researched areas and evidence gaps. Many EGMs indicate the strength or quality of the evidence for each intervention–outcome pair, helping users assess the reliability of available research. Additionally, ours will incorporate whether evidence exists on the mechanisms, moderators, implementation issues, and economics of these SCP interventions against terrorist attacks on soft targets or crowded places.

### 3.2 Framework Development and Scope

The framework for the EGM will be informed by both theory and empirical evidence. Initially, the inclusion and exclusion criteria, along with the search strategies, will be guided by the plan outlined below, shaped by both the academic experiences of the research team and the practical experiences of the Advisory Group.

### 3.3 Stakeholder Engagement

The Department of Homeland Security stakeholders have already reviewed a draft protocol, and feedback was provided before the initiation of the funding formally and informally at three recent in‐person workshops in London, Montreal, and Omaha between March 2024 and August 2024. Once this draft protocol is completed, the same team at DHS will review it. From there, the protocol will be shared with equivalent stakeholders across the Five Eyes network for their feedback, and they will be invited to join an Advisory Group and suggest further members from beyond these government security professions. The Advisory Group will aid in the identification of grey literature, review our included studies, recommend other studies they feel are missing but eligible and provide feedback provision on the initial draft EGM and associated write‐ups. All of the Advisory Group's represented agencies will be listed in the final report.

### 3.4 Conceptual Framework

SCP measures aim to deter or hinder criminals by increasing their perception of the risk of getting caught; increasing the actual risk of getting caught; and denying the opportunity to gain information or access. The presence of situational factors providing guardianship increases the risk of apprehension. Traditionally, target hardening refers to overt efforts to make an area more difficult to target, such as the use of locks, fences, or other security measures. Such measures aim to either make it impossible to approach the potential target or to discourage reconnaissance and planning by offenders due to the elevated risk, effort, or difficulty associated with success (Clarke 2006). There are instances, however, where target hardening measures are intentionally covert to avoid publicizing the location of a potential target through the use of discrete surveillance systems or subtle environmental modifications that increase security without drawing attention to the target. Collectively covert and overt target hardening approaches aim to create an environment where hostile reconnaissance is either rendered ineffective or judged not worthwhile by offenders, thereby enhancing protection without revealing vulnerabilities (Coaffee 2016). Access control refers to measures that seek to allow only legitimate persons to enter an area. This limits permeability and, as such, reduces the opportunity for crime by increasing the effort needed to enter and exit an area. In the case of terrorism, access control could include metal detectors which prevent weaponry from being brought close to specific types of targets. These have been widely recognized as an effective measure in reducing airline hijackings or other types of incidents (Cauley and Im 1988; Enders et al. 1990). Increasing the effort can also include other types of entrance and exit screening, for example, bag checks and pat‐downs.

Formal surveillance works by eliciting a deterrent effect in potential offenders through the deployment of personnel whose primary responsibility is security (e.g., security guards). This is theoretically conceptualized by some scholars as a form of guardianship in SCP (see Hollis‐Peel et al. [2011] for a full discussion on measuring guardianship). An offender's perception of guardianship impacts their perception of the likelihood of being detected. Beyond perception, effective guardianship may increase the actual risk of detection (Welsh and Farrington 2009). Second, the presence of security personnel (often alongside other signs of investment) may have an effect as visible signs of investment. For instance, an area which employs security guards may signal to users of the space that the area is undergoing investment. This may increase users' positive perceptions of the space and thus encourage users to assert greater informal social control over potential offenders (Welsh and Farrington 2009). Third, similarly, signs of investment, including the employment of security personnel, may have a direct deterrent effect upon offenders who perceive the area as ‘improving’. An area undergoing improvement may be more risky to offend within, given the perception of increased controls (both formal and informal) (Taylor and Gottfredson 1986).

Natural surveillance shares the same aim as formal surveillance but involves efforts to ‘capitalize upon the “natural” surveillance provided by people going about their everyday business’ (Clarke 2006, 21). Design for natural surveillance consists of creating opportunities for observation and shifting the focus to members of the public to observe their surroundings. Natural surveillance may also prevent crime by increasing the true probability of being detected. Place managers (Eck and Clarke 2019) are persons such as bus drivers, parking lot attendants, train conductors and others who perform a surveillance function by virtue of their position of employment. Unlike security personnel, however, the task of surveillance for these employees is secondary to their other job duties. Place managers deter potential offenders by increasing their subjective probability of detection.

### 3.5 Dimensions

The dimensions are inspired by both the intervention‐outcome framework and the EMMIE framework. The first ‘E’ of EMMIE refers to ‘effect’ size and covers the first and second objectives of the EGM (above). Typically, this focuses on the ‘effect’ (impact) of an intervention. This is typically expressed in statistical terms, that is, odds ratio, mean differences, and correlation coefficients. The remaining parts of EMMIE will be coded to determine whether evidence exists (y/n) on these issues and cover the third objective of our EGM (see above). The initial ‘M’ refers to the ‘mechanism’ through which an intervention brings about its effect, the way in which an intervention is expected to produce the desired outcome (i.e., reduction in terrorist attacks). The second ‘M’ refers to ‘moderators’ (or ‘contexts’)—the conditions that are instrumental for an intervention to activate the mechanism/s and whether the sought‐after outcome patterns are produced. The ‘I’ refers to ‘implementation’ conditions that support or obstruct the delivery of the intervention. The second ‘E’ refers to ‘economics’, the financial impact of an intervention, in other words, what the intervention will cost in relation to outputs, outcomes or benefits.

Each row will depict an intervention. Interventions will be identified inductively. Columns will be organized according to outcomes/effects (see examples below), moderators, mechanisms, implementation issues, and economic issues. We will examine five types of displacement that could occur subsequent to an intervention: (a) spatial displacement, (b) temporal displacement, (c) target displacement, (d) tactical displacement and (e) perpetrator displacement. The bubble size will reflect the number of studies in that particular domain, and the bubble colour will depict meaningful categories of research methods dictated by the eligible studies. For example, these might include (1) studies using correlation, (2) studies using experimental methods, and (3) studies using qualitative methods.

#### 3.5.1 Types of Study Design

##### 3.5.1.1 Quantitative Studies

Eligible quantitative studies for our EGM will encompass a range of primary studies. At a minimum, quantitative studies must (a) evaluate two or more groups, comparing those with and without the SCP intervention, (b) conduct an analysis of data over time to indicate the temporal ordering of effects, or (c) pre and post‐designs with no control group. We now elaborate upon each.

##### 3.5.1.2 Evaluations of Two or More Groups, Comparing Those With and Without the SCP Intervention

Randomized controlled trials (RCTs) will be included because they are widely considered the gold standard for causal inference due to the minimization of selection bias, and because of their high internal validity. Because preventing terrorist attacks is often a national security priority, RCTs may not always be feasible or ethical when new interventions are deployed. We therefore also include quasi‐experimental designs (QEDs). QEDs maintain our focus on causal inference by leveraging natural experiments, cutoffs or pre‐post intervention data. QEDs that will be eligible for inclusion are as follows:

- Studies where places are assigned to groups non‐randomly before the intervention in a prospective manner.
- Studies, with or without baseline measures, where groups are retrospectively formed based on having previously received the intervention.
- Studies, with or without baseline measures, where groups are retrospectively identified based on the presence of an outcome of interest and then assessed to determine differences in intervention exposure.

##### 3.5.1.3 Analyses of Data Over Time to Indicate the Temporal Ordering of Effects

Here, we include studies that analyze data points collected at consistent time intervals to identify patterns, trends, and potential effects of interventions. These studies might

- Establish baseline patterns before intervention and compare post‐intervention data to the baseline to see if there are significant deviations that can be attributed to the intervention (e.g., ARIMA time series models);
- Establish seasonal patterns before intervention and compare post‐intervention data to see whether the intervention disrupted or altered the seasonal pattern in a meaningful way (e.g., SARIMA time series models);
- Incorporate exogenous covariates such as weather, conflict dynamics, or economic indicators that might impact the outcome and analyze the data to isolate the effect of the intervention from other influencing factors.

##### 3.5.1.4 Pre‐ and Post‐Designs With no Control Group

Studies that explore correlational designs that examine relationships between variables without inferring causation will also be included, although the EGM will note that the evidence from these studies is less robust than those using the aforementioned approaches. We include such designs because they contribute toward a foundational understanding of associations that may warrant further investigation using more sophisticated evaluation techniques, inform theories of change, and help identify mediating factors.

##### 3.5.1.5 Qualitative Studies

We will also seek qualitative evidence from evaluation studies that meet the problem/population criteria stated above. These evaluations may be wholly qualitative in nature or include some quantitative sibling evidence. These studies will provide key evidence on the MMIE strands of EMMIE by providing evidence on implementation issues, contextual factors, and may bridge the gap between causality and contextual understanding. Finally, we will include any relevant systematic reviews because they ensure prior syntheses are leveraged, reduce redundancy, and inform gaps at a higher level.

#### 3.5.2 Types of Intervention/Problem

This EGM will focus specifically on SCP measures that are implemented with the aim of preventing all forms of terrorist attacks, with our searches including all five mechanisms (increase the effort, increase the risks, reduce rewards, reduce provocations, and remove excuses). However, this EGM will focus on the first two mechanisms of SCP: increase the effort and increase the risks, as they are the most relevant to protective security and are the most useful in this context for policy and practice.

Increasing the effort could include interventions such as target hardening, access control, entry and exit screening, deflecting offenders and controlling weapons. Target hardening includes any effort to make an area more difficult to be targeted, for example, the use of locks, fences, and so forth. Access control includes measures designed to prevent offending by restricting legitimate users' access to certain areas. Exit screening refers to frequently used screening techniques such as visual inspection, pat‐downs, metal detector wand use, and walk‐through metal detectors. Deflecting offenders includes the use of wider perimeters of security measures. use the concept of territoriality, which entails ‘the capacity of the physical environment to create perceived zones of territorial influences’ (Newman 1972, 51). Territoriality refers to a sense of ownership over an area. Symbolic territoriality refers to items that signal a change in ownership (e.g., signs and landscaping). Real territoriality refers to physical features that physically impede access to the property (e.g., walls, fences, and gates). Controlling weapons refers to measures designed to prevent the acquisition of weapons.

Increasing the risk includes extending guardianship, assisting natural surveillance, reducing anonymity, utilizing place managers, and strengthening formal surveillance. Formal surveillance is the use of security guards or other employees specifically tasked with watching for offending. The use of closed‐circuit television (CCTV) surveillance cameras is a mainstream SCP measure used around the world in both public and private settings. CCTV cameras serve many functions but are most often used to increase levels of formal surveillance in a target area (Cornish and Clarke 2003; Welsh and Farrington 2009). CCTV's primary aim is to impact offender decision‐making by persuading them to abstain from crime (Ratcliffe 2006). Natural surveillance refers to cases where the legitimate users of the area have the ability to observe what is going on around them without taking special measures, as well as the use of measures such as lighting to increase the ability of individuals to observe what is going on around them.

#### 3.5.3 Types of Population (as Applicable)

We use the Department of Homeland Security definition of *‘Soft Targets and Crowded Places (ST‐CPs)’* to define our population: locations that are easily accessible to large numbers of people and that have limited security or protective measures in place, making them vulnerable to attack. ST‐CPs can include but are not limited to, schools, sports venues, transportation systems or hubs, shopping venues, bars and restaurants, hotels, places of worship, tourist attractions, theatres, and civic spaces. ST‐CPs do not have to be buildings and can include open spaces such as parks and pedestrian malls. ST‐CPs will not necessarily be crowded at all times—crowd densities may vary between day and night, by season, and may be temporary, as in the case of sporting events, festivals, or other special events. This particular definition aligns with several other countries' definitions of soft targets and crowded places^1^ but provides a clearer and more elaborated set of criteria to follow. It is an elaborated version of the UK government's definition but provides greater specificity in certain key criteria. Without this greater specificity, the UK government's definition admits that it allows for more subjectivity (‘what counts as a crowded place is a matter of judgement’), which in turn, would have the potential to negatively impact our coding. This definition is also heavily aligned with the Global Counter Terrorism Forum's definition, which has been accepted by non‐Western countries, including Algeria, China, Colombia, Egypt, India, Indonesia, Japan, Jordan, Kenya, Kuwait, Morocco, Nigeria, Pakistan, Qatar, Russia, Saudi Arabia, South Africa, Turkey and the United Arab Emirates. We operationalize public, semi‐public, and semi‐private places as fitting the definition provided above. Interventions implemented in a location where soft and hard targets are simultaneously present will also be included.

In terms of places, the location must either be

a. Part of the built environment and designed for human congregation (e.g., structures, facilities, and areas).
  OR
b. Natural or civic spaces.

It must also

a. Hold regular or predictable gatherings exceeding 50 individuals per 1,000 square metres during peak times (e.g., crowded place, see introduction to Liu et al. 2024).
  OR
b. Be publicly accessible without substantial barriers (e.g., soft target due to accessibility).
  OR
c. Have limited security or protective measures (e.g., soft target due to lack of protection).

In studies that analyze both hard and soft targets, we will only include studies with over 50% of the targets meeting the above criteria.

#### 3.5.4 Types of Outcome Measures (as Applicable)

Primary outcomes may include the following:

− Number of terrorist attacks.
− Severity of terrorist attacks in terms of fatalities and/or injuries.
− Ability to detect attack planning activities such as hostile reconnaissance or other pertinent antecedents of terrorism (e.g., positive disposals such as arrests and non‐arrest disposal outcomes such as penalty notices).

We will exclude studies that measure the public's perception of such interventions (e.g., the impact on their fear of terrorism) or studies interested in the underlying motivational causes that lead to the execution of such attacks.

#### 3.5.5 Types of Location/Situation

No limits will be placed on the geographical region reported in the study. As per Sydes et al. (2023), we will use Google Translate to ascertain the eligibility of non‐English language documents. If this is not feasible, we will contact the study authors for a determination.

### 3.6 Search Methods and Sources

#### 3.6.1 Database Searches

We will make use of an extensive range of search locations to ensure published and unpublished research is identified across a range of disciplines. To identify studies for the review, we developed a set of terms related to terrorism, which have been synthesized from other Campbell systematic reviews (e.g., Wolfowicz et al. 2020; Lum et al. 2006), others inspired by Campbell systematic reviews (e.g., Gill et al. 2020), the 22 seed articles, and terms related to SCP which have been synthesized from previous systematic reviews undertaken that had a focus upon everyday crimes and that are catalogued in the What Works in Crime Reduction catalogue, as well as Lum et al.'s (2006) systematic review on counter‐terrorism initiatives (see Table 1).

**Table 1:** Core search strategy.

Platform/Database: APA PsycINFO (Ovid)
Search date: 8 December 2024
1. extremism/or exp terrorism/or radical movements/(10255)
2. (‘al‐qaeda’ or ‘al‐qaida’ or anarch* or ‘boko haram’ or bomb or bombed or bomber* or bombing* or bombs or cyberterror* or ‘cyber terror*’ or ‘cyber‐terror*’ or daesh or extremism or extremist or extremists or ‘extreme‐left’ or ‘extreme‐right’ or fanatic* or ‘far‐left’ or ‘far‐right’ or ‘foreign agent*’ or ‘foreign fighter*’ or guerrilla* or hamas or hezbollah or hijack* or incel or incels or indoctrinat* or insurrection* or insurgent* or ISIL or islamis* or jihad* or KKK or ‘ku klux klan’ or klans* or lethal* or lone or loner* or martyr* or militant* or militia* or mujahideen* or neonazi* or ‘neo‐nazi*’ or qanon* or radical* or 4chan or 8chan or 8kun or salafi* or skyjack* or taleban or terror* or violen* or ‘white supremac*’ or zealot*).ti,ab,id.(165328)
3. or/1‐2 (166158)
4. (‘free radical*’ or ‘hydroxyl radical*’ or ‘oxygen radical*’ or ‘peroxide radical*’ or ‘radical prostatect*’ or ‘superoxide radical*’).ti,ab,id.(2421)
5. 3 not 4 (163737)
6. exp crime prevention/(4009)
7. (preven* adj3 (crime* or criminal*)).ti,ab,id. (2886)
8. (barricad* or ‘blast harden*’ or bollard* or ‘card access’ or CCTV or checkpoint* or ‘check point*’ or ‘conceal* target*’ or ‘crime prevention through environmental design’ or CPTED or ‘crowd* limit*’ or ‘crowd size limit*’ or curfew* or ‘detection dog*’ or detector* or ‘entry gate*’ or fence* or fencing or guardian* or lighting or ‘limit* crowd*’ or ‘perimeter security’ or ‘physical security’ or ‘place manager*’ or ‘polic* patrol*’ or ‘public reporting’ or ‘reduc* crowd*’ or ‘remov* target*’ or ‘ring of steel’ or SCP or ‘secur* by design’ or ‘security measur*’ or ‘security patrol*’ or ‘secur* perimeter*’ or ‘situational crime’ or ‘situational prevention’ or ‘sniffer dog*’ or ‘stop and search*’ or surveil* or ‘target harden*’).ti,ab,id.(33884)
9. (control* adj2 (access or entries or entry or exit* or tool* or weapon*)).ti,ab,id. (1476)
10. counterterrorism/(509)
11. (countermeasur* or ‘counter‐measur*’ or ‘preventive measur*’ or ‘security measur*’).ti,ab,id.(6406)
12. ((counterhijack* or ‘counter‐hijack*’ or counterinsurgen* or ‘counter‐insurgen*’ or counterintelligen* or ‘counter‐intelligen*’ or counterradicali* or ‘counter‐radicali*’ or counterterror* or ‘counter‐terror*’) adj5 (interven* or measur* or plan* or program*)).ti,ab,id. (113)
13. ((defeat* or deter* or disrupt* or dissuad* or limit*) adj4 (atrocit* or extrem* or hijack* or skyjack* or ‘soft target*’ or terror* or ‘vulnerable target*’)).ti,ab,id.(1834)
14. ((‘pre‐caution*’ or precaution* or ‘pre‐empt*’ or preempt* or preven* or reduc* or stop*) adj4 (attack* or atrocit* or bomb* or extrem* or hijack* or skyjack* or ‘soft target*’ or terror* or ‘vulnerable target*’)).ti,ab,id.(2400)
15. ((bag* or passenger* or security or traveller* or traveller*) adj3 screen*).ti,ab,id. (213)
16. or/6‐15 (50444)
17. 5 and 16 (5327)
18. animal.po. (451124)
19. exp animals/or animal models/or exp animal research/or exp primates/(400653)
20. or/18‐19 (467930)
21. human.po. (4840642)
22. 20 not 21 (401317)
23. ((afrik* or alban* or arab* or bulgar* or catalan or chinese or croat* or cze* or danish or dut* or estonian or fars* or finn* or french or georgian or ger* or gre* or hebr* or hindi or hungarian or ital* or japan* or kor* or latv* or lith* or malay* or nonenglish or norweg* or polish or portug* or roman* or rus* or serb* or slovak or sloven* or spa* or swed* or turk* or ukr* or urdu) not english).lg. (343111)
24. 17 not (22 or 23) (5093)
25. limit 24 to yr = ‘2000–Current’ (4663)

We will search for the above sets of terms across title, abstract, author‐supplied keywords, and indexing term search fields for documents published from January 2000 until October 2024. The 22 seed articles informed the specific search term words. The starting point of the year 2000 is deemed appropriate for this EGM based on the findings of Lum et al.'s (2006) systematic review of counter‐terrorism evaluation research. Just five studies were published before 2000 and either evaluate counter‐terrorism measures other than SCP (e.g., Landes 1978; Brophy‐Baermann and Conybeare 1994), evaluate multiple measures (some SCP, some not), which renders isolating the effects of SCP measures difficult, or model for a wide range of targets and do not isolate for crowded places and soft targets (e.g., Enders et al. 1990; Enders et al. 1990; Cauley and Im 1988). The following academic databases will be searched: APA PsycINFO (Ovid), APA PsycEXTRA (Ovid), Applied Social Sciences Index & Abstracts (ASSIA) (ProQuest), Australian Criminology Database (CINCH) (Informit), Criminal Justice Abstracts (EBSCO), Dissertations & Theses Global (ProQuest), Global Policing Database (UQ), International Bibliography of the Social Sciences (IBSS) (ProQuest), International Political Science Abstracts (EBSCO), Scopus (Elsevier), Sociological Abstracts (ProQuest), Social Services Abstracts (ProQuest), WoS Book Citation Index – Social Sciences & Humanities (BCI) (Clarivate), WoS Conference Proceedings Citation Index – Social Sciences & Humanities (CPCI‐SSH) (Clarivate), WoS Emerging Sources Citation Index (SSCI) (Clarivate), WoS Social Science Citation Index (SSCI) (Clarivate). These were chosen based on scans of previous adjacent systematic reviews as well as a pilot test of our search strategy that retrieved all 22 seed articles that look relevant to the goals of our EGM.

Searching Other Resources

In addition to the database searches, we will search through the following:

− Relevant government and non‐government websites related to terrorism research (e.g., Centre for Counter‐Terrorism Coordination (https://www.nationalsecurity.gov.au/Pages/default.aspx), Centre of Excellence Defence Against Terrorism (https://www.coedat.nato.int/research.html), Combating Terrorism Centre (https://ctc.westpoint.edu), CREST (https://crestresearch.ac.uk), Department of Homeland Security (https://www.dhs.gov), ICCT (https://icct.nl/publications), NaCTSO (https://www.protectuk.police.uk), National Institute of Justice (https://nij.ojp.gov/library), National Protective Security Authority (https://www.npsa.gov.uk/learning-resources), Naval Postgraduate School, NCITE (https://www.unomaha.edu/ncite/index.php), Public Safety Canada (https://www.publicsafety.gc.ca/index-en.aspx), START (https://www.start.umd.edu), United Nations Office of Counter‐Terrorism (https://www.un.org/counterterrorism/resources)).
− Forward citation searches on eligible studies using the ‘Cited By’ function on Google Scholar, manually reviewing titles and abstracts for studies that appear eligible, and then reviewing their full texts against our previously stated eligibility criteria.
− Backward citation searches on eligible studies.
− Advisory group inputs to identify any further missing studies.
− Trial registers for ongoing studies (which will be included in the report but not the map). The following trial registries will be searched:
  ∘ The American Economic Association's registry for RCTs (https://www.socialscienceregistry.org).
  ∘ National Institutes of Health RePORTER (https://reporter.nih.gov).
  ∘ World Health Organization International Clinical Trials Registry Platform (https://trialsearch.who.int).
  ∘ Trials Register of Promoting Health Interventions (https://eppi.ioe.ac.uk/webdatabases4/Intro.aspx?ID=12).
  ∘ EU Clinical Trials Register (https://www.clinicaltrialsregister.eu).
  ∘ NHS Health research Authority (https://www.hra.nhs.uk/planning-and-improving-research/research-planning/research-registration-research-project-identifiers/).

### 3.7 Analysis and Presentation

#### 3.7.1 Report Structure

The EGM will comprise an executive summary, background, objectives, methods, results and discussion.

The executive summary will comprehensively outline the EGM findings and highlight the number of studies included in the review. The background section will address the issue of countering terrorist attacks and explain why and how situational approaches may help disrupt and prevent their occurrence and impact. The methods section will outline the systematic search process, including the key search terms and the databases or sources consulted. It will also describe the screening procedures, data extraction methods, and the software used throughout the process. The results section will summarize the number of eligible studies identified in the systematic search, presented in a PRISMA flow diagram. Additionally, the results will feature our interactive EGM, offering a visual depiction of the available evidence. The discussion will consider the distribution of research across various intervention and outcome areas, noting both gaps and concentrations. We will provide recommendations for future research and synthesis, and discuss the implications for policy and practice. We anticipate including one figure (PRISMA) and four tables (search locations including all grey literature; non‐interactive EGM of primary studies including grey literature; online interactive EGM—with filters).

#### 3.7.2 Filters for Presentation

The results of our online interactive EGM will be presented as a matrix. Each row will depict an intervention. Columns will be organized according to outcomes/effects, moderators, mechanisms, implementation issues, and economic issues. An additional column will be used to illustrate any displacement or diffusion of benefits resulting from the intervention that has been identified. We will also use the following filters:

- Study type (toggle filter): research study, research synthesis.
- Research design: correlational, RCT, quasi‐experimental.
- Study location: country, region.
- Ideology of targets: mixed, Islamist, extreme‐right wing, ethno‐nationalist, extreme‐left wing.
- Document type: published, unpublished, peer‐reviewed, grey literature.

#### 3.7.3 Dependency

Regarding dependency, we follow the process laid out by Sydes et al.'s (2023) EGM on criminal justice interventions in the prevention of violent extremism (paraphrased below). The unit of analysis in this context is the individual study rather than the publication or document that presents the study's findings. It is important to recognize the potential issue of using documents as the unit of analysis, as visual tools like an Evidence Gap Map (EGM) may give the impression of an abundance of evidence for an intervention when multiple publications are based on the same study.

In an EGM, dependency typically manifests in two main ways: either as a ‘many‐to‐one’ or a ‘one‐to‐many’ relationship. The ‘many‐to‐one’ dependency occurs when several documents report on the same study, in which case these documents are linked together, and the study itself is displayed on the map. In contrast, the ‘one‐to‐many’ dependency happens when a single document reports on multiple studies, in which case the document is linked to each of the studies it covers. Systematic reviews pose a distinct challenge regarding the ‘one‐to‐many’ dependency, as they often include multiple studies that address different interventions and outcomes. The difficulty lies in determining how to represent a systematic review in an EGM (note in this instance, none currently exist in the research literature that we are aware of). If a systematic review is shown alongside its component studies, it may give a misleading impression of the volume of evidence. On the other hand, if only the component studies are displayed, the EGM would fail to indicate where studies have been synthesized rigorously or where attempts at synthesis, such as in empty reviews, have been made. To address this, we propose a multi‐pronged approach for systematic reviews in the EGM should any be found or published:

1. We will extract the primary studies from each systematic review and link the documents back to their respective parent studies, just as we would for any ‘one‐to‐many’ relationship.
2. We will include systematic reviews in the EGM but separate them from primary studies by using a toggle filter for study type, with mutually exclusive options for research syntheses and individual studies. This will allow users to switch between a map of primary studies and a map of systematic reviews, ensuring that the volume of evidence is not visually overstated.

Additionally, there is another type of dependency where a single study compares two eligible treatments directly. In such cases, the study might need to be represented in two different sections of the map. To address this, we will not only link related documents but also include an identifying maker on studies of this design to highlight the potential for double‐counting. Similarly, if either (a) a systematic review qualifies for more than one category on the map, it will be flagged to alert users to the risk of double‐counting, or (b) a study qualifies for more than one of the EMMIE columns on the map, it will be similarly flagged to alert users.

### 3.8 Data Collection and Analysis

#### 3.8.1 Screening and Study Selection

We will first conduct a training phase of the screening process. Three researchers will screen 100 randomly selected records. A Fleiss Kappa > 0.80 will be required before the formal screening process begins. If the IRR is below the threshold, a consensus meeting will take place, followed by a repeat training exercise of 50 randomly chosen records. Another Fleiss Kappa IRR will be run. If it is > 0.80, formal screening will resume. If it is < 0.80, a repeat procedure of a consensus meeting and repeated training exercise will continue until a Fleiss Kappa > 0.80 is achieved.

Screening will be conducted in two stages: (a) title and abstract, followed by (b) full text. Two researchers will screen each title/abstract as well as each full text. A third party will be involved in the case of disagreements which cannot be resolved through discussion.

*Title and Abstract Screening*

This data will be assessed on the title and abstract on the following criteria:

1. Document is not unique.
2. Ineligible document type.
3. Document is not place‐based.
4. Document does not regard a counter‐terrorism intervention for preventing terrorist attacks.

Before screening, we will endeavour to remove any duplicates and ineligible document types (such as, blog posts, book reviews, etc.). Criteria 1 and 2 will allow us to remove any that were missed during data cleaning. Titles and abstracts unrelated to terrorism will be removed as per Criterion 3.

Systematic searches often result in several thousand results, which traditionally are manually sifted by human researchers. Doing so can be prone to error, given the demanding nature of the task. It is also time‐consuming and costly in terms of resourcing. As such, tools designed to improve the efficiency of the review process have begun to emerge. In our case, references obtained from databases and grey literature searches will be subject to quasi‐automated sifting via ASReview (van de Schoot et al. 2020; de Boer et al. 2021). ASReview uses human inputs to ‘learn’ about which texts are more relevant to the inclusion criteria to organize results from most relevant to least relevant. The idea is that once sufficient manual screening and (machine) learning have occurred, there is no need for researchers to sift the irrelevant records. ASReview has been independently validated as an efficient screening tool (Chan et al. 2024), and can reduce screening burden by up to 82% (Ferdinands et al. 2021). ASReview is free, open source, and transparent (i.e., all code to execute ASReview is freely and openly available for public scrutiny). Hence, ASReview will be used for the initial sift on Title and Abstract.

To train ASReview, during the preparation phase, we will label the aforementioned 22 seed studies as relevant for full‐text review and an equivalent number of other studies as not relevant for full‐text review. Initially, we will use Naïve Bayes as the machine learning model, Naïve Bayes is preferable in situations (a) where there is a very limited number of labelled examples and (b) within initial phases of active learning. Naïve Bayes will work alongside uncertainty sampling as an active learning strategy to identify which batches of articles require reviewing next. After each article is labelled, the model updates and learns from the coding and re‐sorts its next batch of suggested articles to code. Because we are using seed articles to train the model, we expect ASReview to find and prioritize relevant studies for coding quickly, and over time the rate of new relevant studies will slow as the model learns.

ASReview provides several heuristics to inform and determine your stopping criteria. This sift will use an inclusion rate of 0.1% per batch of 1000 records—meaning if a batch of 1000 records is consecutively coded as not relevant, we will stop the sift. Once we have reached the stopping criteria, we deploy two strategies to ensure the robustness of the search. First, we will randomly sample sets of 20 uncoded records to ensure no new relevant records are found. We will conduct these random samples until five consecutive sets reveal no new relevant records (Sydes et al. 2023). Second, we will deploy a Support Vector Machine as the new machine learning model, which is better suited for later phases of active learning when many more labelled examples are available, allowing for more complex and precise decision boundaries. Again, we will sift until 1000 consecutive non‐relevant records have been coded.

The additional strategies outlined elsewhere (e.g., Advisory group inputs, forward/backward citation searches) will also mitigate any potential missing articles. There will be a point where the research team can determine with some confidence that all remaining texts are irrelevant and, therefore, do not require manual review.

*Full‐Text Screening*

Once the screening of the title and abstract is completed, full‐text searches will be conducted. The documents/PDFs that made it through this initial screening will be imported into EPPI 6 Reviewer—a web‐based tool for managing and conducting systematic reviews. The full‐text of the records will be screened according to the following exclusion criteria:

1. Document is not unique.
2. Ineligible document type.
3. Document is not about a counter‐terrorism intervention.
4. Document is not about a SCP intervention.
5. Document is not about an SCP intervention to prevent terrorist attacks.
  in crowded places/against soft targets (explicitly stated by the authors).
6. Document does not report data which may be indicative of a process or impact evaluation.

Criterion 3 will refine the body of studies to those that may be indicative of an evaluation. Criterion 4 will remove records not pertaining to terrorism. Criterion 5 will limit us to studies that report on evaluations of SCP interventions using eligible research designs.

EPPI's machine learning‐powered search function, OpenAlex, will be used to supplement searches. OpenAlex uses training data (your included studies) to search a database for any likely related studies. This may highlight any thus far unidentified texts. The same inclusion criteria will be applied to full texts and any texts identified by OpenAlex until we have a final sample of studies for the EGM. Furthermore, we will import all records identified through our grey literature search and later conduct forward/backward citation searches into OpenAlex.

#### 3.8.2 Data Extraction and Management

EPPI‐reviewer will be used to code eligible documents across: interventions, effects, moderators, mechanisms, implementation, economics, displacement/diffusion of benefits, research methodology, and population. Two coders will code all eligible studies and check for inter‐rater reliability. Authors will be contacted if key information is missing.

##### 3.8.2.1 Tools for Assessing Risk of Bias/Study Quality of Included Reviews

A validated risk of bias tool will be applied to all included studies. This is a form of critical appraisal used in systematic reviews to assess the quality of included studies. The features of included studies are examined to identify if any aspects of the design or conduct of the study could lead to unreliable or misleading results. A final judgement can be made about whether to include or exclude a study, or quality assessment can be used to identify studies which may be more or less reliable. Which tool to use will be determined by the nature of the studies we extract. In some instances, it is appropriate to modify a validated risk of bias tool to meet a specific project's needs—we may elect to do so.

For RCTs, we will use the Cochrane Risk of Bias Tool (RoB 2), which evaluates domains like randomization, deviations from the intended intervention, missing outcome data, and measurement of the outcome. For QEDs and time‐series analyses, we will use the Risk of Bias in Non‐Randomized Studies—of Interventions (ROBINS‐I) which covers domains such as confounding, selection bias, classification of interventions, deviations, missing data, and measurement outcomes. For correlational cross‐sectional studies, we will use the JBI Checklist for conducting correlational cross‐sectional studies to assess the risk of bias.

Assessing the strengths and limitations of single pieces of qualitative research remains highly contested (Garside 2014). We will therefore use the Critical Appraisal Skills Programme (CASP) tool, which is endorsed by the Cochrane Qualitative and Implementation Methods Group and is ‘the most commonly used checklist/criteria‐based tool for quality appraisal in health and social care‐related qualitative evidence syntheses’ (Long et al. 2020, 32), and looks highly applicable to this domain. The CASP tool comprises a 10‐question checklist to interrogate each qualitative study concerning: the clarity of its research statement; the appropriateness of the qualitative methodology, the research design, and the recruitment strategy; the data collection process, positionality, ethical issues, the rigour of the data analysis, the clearness of the findings, and the value of the research. We will follow Long et al.'s (2020) guidance on using the CASP tool for identifying higher‐quality studies.

For systematic reviews of SCP measures against terrorist attacks at soft targets or crowded places, we will use the AMSTAR II checklist to assess methodological quality (Shea et al. 2016). AMSTAR II has been found to be a valid and reliable appraisal tool (Lorenz et al. 2019; Gates et al. 2018; Pieper et al. 2019). AMSTAR II comprises 16 items.^2^ The 16 items are broken into two types: critical and non‐critical. If a critical item is not present, it is considered a flaw. There are 7 critical items.^3^ If a non‐critical item is not present, it is considered a weakness. There are 9 non‐critical items.^4^ The results provide a rating on the overall confidence in a review's results. Potential outcome ratings range from high to critically low:

− High – Zero or one non‐critical weakness: The systematic review provides an accurate and comprehensive summary of the results of the available studies that address the question of interest.
− Moderate – More than one non‐critical weakness: The systematic review has more than one weakness but no critical flaws. It may provide an accurate summary of the results of the available studies that were included in the review.
− Low – One critical flaw with or without non‐critical weaknesses: The review has a critical flaw and may not provide an accurate and comprehensive summary of the available studies that address the question of interest.
− Critically low – More than one critical flaw with or without non‐critical weaknesses: The review has more than one critical flaw and should not be relied on to provide an accurate and comprehensive summary of the available studies.

##### 3.8.2.2 Methods for Mapping

EPPI‐Mapper will be used to provide an interactive and freely accessible visual map.

## Author Contributions

Content: Zoe Marchment, Paul Gill. EGM methods: Zoe Marchment, Caitlin Clemmow, Paul Gill. Statistical analysis: Zoe Marchment, Caitlin Clemmow. Information Retrieval: Zoe Marchment, Caitlin Clemmow, Paul Gill, Doug Salzwedel.

## Conflicts of Interest

The authors declare no conflicts of interest.

## Plans for Updating the EGM

Updates are anticipated to occur every 5 years.

## Supporting information


**Appendix 1. Coding template**.

## Endnotes

1 See Australia: https://www.nationalsecurity.gov.au/crowded-places-subsite/Files/crowded-places-security-audit.pdf; See UK: https://assets.publishing.service.gov.uk/media/5a7df5dded915d74e6223310/Crowded_Places-Planning_System-Jan_2012.pdf.

2 For a full accounting of AMSTAR, see https://amstar.ca/docs/AMSTAR%202-Guidance-document.pdf.

3 (1) Protocol registered before the commencement of the review; (2) Adequacy of the literature search; (3) Justification for excluding individual studies; (4) Risk of bias from individual studies being included in the review; (5) Appropriateness of meta‐analytical methods; (6) Consideration of the risk of bias when interpreting the results of the review; (7) Assessment of presence and likely impact of publication bias.

4 (1) Research questions and inclusion criteria used PICO components; (2) Study design selection reported; (3) Study selection performed in duplicate; (4) Extraction performed in duplicate; (5) Adequate description of included studies; (6) Funding sources reviewed; (7) Risk of bias considered in results of meta‐analysis; (8) Sufficient explanation for any heterogeneity observed; (9) Conflict of interests reported or not.
