# Supplementary material for: PROTOCOL: Situational Crime Prevention Measures to Prevent Terrorist Attacks Against Soft Targets and Crowded Places: An Evidence and Gap Map
Source: Campbell Syst Rev. 2025 Apr 28;21(2):e70040. doi: 10.1002/cl2.70040 (PMC12037697; doi:10.1002/cl2.70040)
Supplement: Supplementary file 1 — Appendix 1. Coding template. [file CL2-21-e70040-s001.docx]

**Appendix 1**

**Coding template**

**Study Details**

1. Study ID [textbox]
2. Author [textbox]
3. Date [textbox]
4. Reference [textbox]
5. Type of document [dropdown menu]
6. Peer-reviewed journal article
7. Book chapter
8. Dissertation
9. Government report, technical report, or working paper
10. Conference presentation
11. Other [specify in textbox]
12. Type of study [dropdown menu]
13. Research study
14. Research synthesis
15. Publication status [dropdown menu]
16. Published (peer reviewed)
17. Published (non-peer reviewed)
18. Unpublished

**Intervention**

1. Location of intervention (city, state/province/county, country) [textbox]
2. Type of intervention [textbox]
3. Year of intervention implementation [textbox]
4. Method [textbox]
5. Ideology of targets [checkbox]
6. Islamist
7. Extreme right wing (XRW)
8. Extreme left wing
9. Single issue
10. Ethno-nationalist and separatist
11. Mixed, unstable, unclear
12. Population [checkbox]
13. Schools/colleges/universities
14. Sports venues
15. Music venues
16. Transport hubs
17. Shopping venues
18. Bars and restaurants
19. Hotels/accommodation
20. Places of worship
21. Tourist attractions
22. Theatres
23. Parks
24. Civic spaces
25. Other [textbox]

**EMMIE**

1. Outcomes/effects reported [dropdown menu]
2. Yes
3. No
4. Outcomes/effects details [textbox]
5. Moderators [dropdown menu]
6. Yes
7. No
8. Moderators details [textbox]
9. Mechanisms [dropdown menu]
10. Yes
11. No
12. Mechanisms details [textbox]
13. Implementation [dropdown menu]
14. Yes
15. No
16. Implementation details [textbox]
17. Economic [dropdown menu]
18. Yes
19. No
20. Economic details [textbox]

**Unintended Outcomes**

1. Displacement examined [dropdown menu]
2. Yes
3. No
4. Displacement found [dropdown menu]
5. Yes
6. No
7. Displacement type [checkbox]
8. Spatial
9. Temporal
10. Target
11. Tactical
12. Perpetrator
13. Displacement details [textbox]
14. Diffusion of benefits examined [dropdown menu]
15. Yes
16. No
17. Diffusion of benefits found [dropdown menu]
18. Yes
19. No
20. Diffusion of benefits details [textbox]
